# Second Chances: Investigating Athletes’ Experiences of Talent Transfer

**DOI:** 10.1371/journal.pone.0143592

**Published:** 2015-11-24

**Authors:** Áine MacNamara, Dave Collins

**Affiliations:** Institute of Coaching and Performance, University of Central Lancashire, Preston, United Kingdom; University of Rome, ITALY

## Abstract

Talent transfer initiatives seek to transfer talented, mature individuals from one sport to another. Unfortunately talent transfer initiatives seem to lack an evidence-based direction and a rigorous exploration of the mechanisms underpinning the approach. The purpose of this exploratory study was to identify the factors which successfully transferring athletes cite as facilitative of talent transfer. In contrast to the anthropometric and performance variables that underpin current talent transfer initiatives, participants identified a range of psycho-behavioral and environmental factors as key to successful transfer. We argue that further research into the mechanisms of talent transfer is needed in order to provide a strong evidence base for the methodologies employed in these initiatives.

## Introduction

As standards in performance sport rise ever higher, coaches, sports, and even athletes are searching for new avenues for recruitment. One recently emerged idea is “Talent Transfer” (hereafter TT), the more or less structured transfer and fast-tracking of talented individuals from one sport to another sport where there are opportunities to succeed. By transferring athletes from donor sports into targeted sports the probability of identifying athletes with the capabilities to compete at the highest level is thought to be increased by minimising adolescent maturational issues (cf. the substantial literature on the relative age effect) [1] and maximising the developmental investment already made in these older athletes [2,3]. In the UK, since 2007, over 7000 individuals have applied to several targeted TT programmes offered by UK Sport with 100 athletes progressing into the World Class system. These results suggest that there is considerable merit in TT initiatives that provide opportunities for athletes to maintain their involvement in elite sport as well as pragmatically targeting sports where success on the world-stage may be more attainable [4, 5, 6, 7]. While there is little contention that TT is a viable means of identifying ‘mature’ talent, however, formal TT appears to be no more effective or efficient in converting ‘talent’ than informal processes or spontaneous transfer [8]. Of course, informal TT has been occurring for some time, when abilities are noticed by coaches or through an athlete looking for a new challenge. However, formal TT initiatives, the systematic selection of athletes based on defined protocols, are proposed to provide a more effective approach to this process [8].

In an era of increasing accountability it is surprising, given the significant financial investment into TT internationally, that there is a lack of rigorous scientific exploration into the mechanisms underpinning the approach, the ‘cost-benefit’ of these initiatives against other options, or even the potential for different and more efficacious variants. The veracity of formal TT is often justified by high profile success, while the dearth of peer reviewed evaluation is explained by the need for maintaining competitive advantage (personal communication, UK Sport). This stands in stark contrast to the government emphasis on evidence-based practice in other areas such as education [9] and medicine [10]. In fact, two of the very few papers that address this topic [4, 7] do little more than describe the recruitment rates and processes involved in the TT process. Although Collins and colleagues (2014) provide some insight into the mechanisms underpinning TT, further understanding of how an athlete’s past experience of learning and performance influence the new sport and the manner by which they adapt this to novel situations [11] would be beneficial; in short, understanding the factors that athletes’ perceive to aid TT should surely enable positive refinement of the process. The extent to which past experiences, skills and knowledge facilitate and optimize the TT process is certainly worthy of investigation as explicit consideration of these factors may well improve the sophistication of TT initiatives.

Typically, TT initiatives screen a large number of athletes based on performance (i.e., athletic background, sporting history) and anthropometric (UK Sport’s Talent 2016: Tall and Talented campaign, for example, has targeted athletes aged between 15 and 22 who are over 190cm tall for men and 180cm tall for women) variables before inviting selected athletes to a testing day where they complete a number of physiological and performance tests. Sporting Giants, for example, was the first TT initiative developed by UK Sport in 2007 and nearly 4000 athletes were first selected based on meeting three criteria (i.e., between 16–25 years of age, over 6’3 for males and 5’11 for females, and an athletic background) and then assessed using a battery of tests including anthropometric (e.g., height, weight, arm span), power (e.g., vertical jump), speed (e.g., 5m, 10m, & 20m sprints), endurance (e.g., multistage fitness test), and skill (e.g., sport specific motor coordination tasks) assessments designed against profiles of elite athletes in the targeted sport. Following this assessment phase, a smaller cohort of athletes (*n* = 58) was selected to attend a “talent confirmation phase” in sports thought to suit their physiological and anthropometric profile (i.e., handball, rowing, volleyball, and canoeing). It is only at this stage of the TT testing process that psychological assessment and behaviour/personality screening as well as sport specific instruction takes place [5, 6, 12].

The empirical evidence and theoretical basis of the initial screening and selection processes described above can be questioned in a number of ways [13]. First, the notion that “talent” can be identified using once-off anthropometric, physiological, and performance measures has been comprehensively questioned in the literature, although the focus has been primarily on young athletes [14]. Nonetheless, we would argue that this approach to talent identification is reductionist and narrow and fails to appreciate that excellence is not idiosyncratic to a specific set of skills or physical attributes but rather, can often be achieved through unique though idiosyncratic combinations of skills, attitudes, and behaviors [14, 15]. In this manner, talent is understood to emerge from “complex and unique choreographies” between different groups of causal influences [16]. Thus, there is clearly not a single genetic endowment underlying a talent domain, with neither talented nor untalented individuals emerging from genetically homogenous groups [17]. Accordingly, these “snapshot” screening procedures may well overlook athletes who do not at the time of testing meet prescribed physical, performance or anthropometric standards, or predetermined profiles, but who may have the potential to develop in the future.

As a second consideration, it is unclear exactly what factors, or more likely combination of factors (cf. multiplicative factors) [17], genuinely and demonstrably underpin successful transfer (i.e., achieving success as World / Olympic level). In some cases (e.g., sprinting to bobsleigh), the potential for transfer is obvious and the transfer between sports has a high degree of face validity. Other transfers appear to have no obvious underlying rationale however, with little overlap apparent between the two sports, apart from the generic, psychosocial components associated with being a successful athlete (cf. Grit–[18]; Psychological Characteristics and Development of Excellence [PCDEs]–[19, 20]). As such, an exploration of the range of factors athletes’ perceive to support TT would seem a logical step in providing an evidence base for applied initiatives or even improving their design and efficiency.

Reflecting these issues, and against the growing literature which highlights positive developmental characteristics, it seems a likely conjecture that TT is facilitated by the identification, development, and promotion of transferable, complementary and interactive elements (i.e., motor, physiological, perceptual, conceptual, physical, psycho-behavioral) in both donor and recipient sports [16, 20]. Indeed, a recent examination [8] questions the methodology of formal TT identification protocols where psychosocial variables are either largely ignored or undervalued, certainly during initial selection. In contrast, a positive and empirically grounded feature of *some* TT programmes is the inclusion of a talent confirmation phase where selected athletes spend time in the sport system with a view to assessing how they cope with training and environmental requirements [5]. The validity of this approach is well supported in the literature, stressing the importance of representative design during the selection process by employing tasks that are representative simulations of the performance environment; for example, tasks that represent variability, ensure decisions are context dependent, and consider individual differences. As such, transferees must have the skills and support (largely psychosocial; [19, 20]) needed to successfully cope with these novel challenges. Furthermore, the role of psycho-behavioral factors [21] in predicting career success is well established suggesting that TT initiatives should consider whether potential transferees have the psycho-behavioral skills needed to successfully develop in the transfer environment [22, 23]. Unfortunately, the initial screening of athletes does not account for these process markers [2] and it is only in the talent confirmation phase, reached by a minority of athletes in formal TT processes, where these factors are tested [6]. Of course, there are strategic and resource implications to any talent identification model; in a practical sense, and reflecting resource issues, formal TT processes involve selecting those athletes who are most likely to be successful and from a pragmatic perspective this will involve some inclusion and exclusion error. However, examining and understanding the range of factors athletes’ perceive as facilitating the TT process would seem a logical starting point to guide formal initiatives and maximize the investments made. As such, learning from the experiences of TT athletes represents a useful starting point for this process. Indeed, the extent to which the transfer environment differs from typical high performance environments is also an under-researched, though potentially key, aspect of TT. It may well be that the “transfer” coach has a significant role to play in facilitating TT, as s/he does at other stages of talent development [24] and therefore attention to this facet of TT warrants attention.

Against these concerns, and before Vaeyens and colleagues’ (2009) suggestion that organized TT reduces “uncertainty in talent identification” (p. 1377) can be adopted with confidence, it is important that the mechanisms underpinning TT are explored. Therefore, the purpose of this exploratory study was to identify the factors which successfully transferring athletes cited as facilitative of effective transfer from one sport to another at the elite level.

## Method

### Participants

Data were collected from seven elite, individual sport athletes (5 female, 2 male) whose mean age was 36.5 years (SD = 6.7 years). All participants had successfully transferred from an elite level in the donor sport (defined as participation at a global level) to the equivalent level in the transfer sport (see Table 1). Five out of the seven participants had medaled at international level (e.g., Olympic Games, World Championships, European Championships, Commonwealth Games) in both sports; details of their performances have not been presented, however, in order to maintain individual confidentiality. As such, this group represented a high level sample from a small and select group of athletes. None of the participants had completed the transfer as part of a formal TT process but instead the transfer was either self or coach initiated. In all cases, the transfer was seamless and there was no overlap between the donor and target sport participation.

**Table 1:** Participant Information^1^.

Participant	Donor sport	Transfer sport	Age of Transfer
1	Track and Field	Water-based Sport	25–30
2	Horse Racing	Individual Power Sport	25–30
3	Individual Power Sport	Multi-sport	25–30
4	Water-based Sport	Individual Power Sport	25–30
5	Water-based Sport	Individual Power Sport	30–35
6	Combat Sport	Combat Sport	30–35
7	Combat Sport	Combat Sport	30–35

^1^ Several participants achieved medals at the highest levels in their new sport. This information is withheld to protect anonymity.

### Procedure

Ethical approval was granted from The University of Central Lancashire’s Built Environment, Business, Arts, Humanities and Social Sciences (BAHSS) Research Ethics Committee and informed consent was obtained from all participants, who were recruited by personal contact. All participants were provided with a written information sheet explaining the study and written informed consent was obtained from all participants.

A semi-structured interview methodology was adopted which began with an introduction explaining the purpose of the study along with assurances of confidentiality. All interviews were conducted by the first author. In an effort to overcome some of the limitations of retrospectively reported information, it was crucial that each participant was able to relate his or her experiences to the key stages and events of their own career and talent transfer process [25]. This approach has been previously shown to increase the accuracy and veracity of recall [26]. Accordingly, in the first phase of the interview each participant was asked to plot their sport career graphically and highlight significant events, turning points, and critical episodes/learning points that influenced their trajectory. Following this, in the second stage of the interview, guided questioning enabled an exploration of the factors that facilitated the talent transfer process. This process allowed the talent transfer experience to be mapped out for each individual and provided a framework in which participants would respond accurately and thoroughly about their own experiences [25]. Ensuring that the participants were asked about specific events, stages, and transitions was another important step towards increasing the validity of the responses [26]. This line of enquiry forced the participant to infer and reconstruct answers to general questions and thus addresses the validity of the responses [27]. Probes and prompts were used to aid clarification and elaboration [28]. The key interview questions during this phase were:

- Based on your timeline, tell me about your background in sport?
- Why did you decide to change sport? Why did you choose [transfer sport]?
- Tell me about your initial experiences in [transfer sport]? What factors facilitated the transfer process? What factors hindered the transfer process?
- Tell me about your progress in [transfer sport]. What factors helped? What factors hindered your transition? What would have helped make the transition smoother?

### Data Analysis

All interviews were transcribed verbatim. Each interview lasted between 50 and 105 minutes, supported by introductory and debrief phases. Data were analyzed using the qualitative data analysis program Atlas.ti. An inductive approach was taken in the analysis of the data [29]. Participants’ quotations were used to depict the lower order themes that formed the first level of analysis of the data. Once these statements were compiled, an inductive analysis of the data was undertaken to generate higher-order themes that linked similar raw data themes together into a higher-order concept. A constant comparative process allowed for the continuous refinement of the results throughout the analysis process until theoretical saturation was met [30]. This involved comparing different participants with each other, comparing the same participant at different times during the interview, comparing data within a higher-order category, and data between lower-order categories.

#### Addressing Trustworthiness

It was important that steps were taken to optimize data trustworthiness [31]. In the first instance, trust and rapport with interviewees was established by the first author, an experienced qualitative researcher and applied practitioner, via an appreciation of their individual history, their current situation and the demands of the TT experiences. Validity of responses was checked using respondent validation techniques [28] which involved follow-up meetings with each individual to discuss the emerging results and the accuracy of the quotations attributed to each individual. Following this, no categories were changed and the exemplar quotations were all supported by the participants.

Trustworthiness of the analytical process was also addressed. A collaborative approach was taken during the analysis stage that involved a constant comparative approach and the challenging of data interpretation by all members of the research team that ensured that evolving meaning was continually re-evaluated and re-asserted. Where alternative explanation and questions over accuracy or potential bias occurred, reflective discussion took place until all themes and their location in the thematic hierarchy were agreed [31]. Recognizing the potential for interpretative bias, and in an effort to ensure rigor, a reflexive journal was also maintained during the data analysis phase [28].

## Results and Discussion

The purpose of this study was to identify the factors which successfully transferring athletes cite as facilitative of effective transfer from one sport to another at the elite level. Two higher order themes were found to facilitate this process. The first section outlines the factors identified as key within the TT environment (Table 2). Following this, the individual factors cited as facilitative of successful transfer from one sport to another are presented (Table 3). Exemplar quotations are used throughout to promote an appreciation of the context in which the themes were generated. All the higher-order themes presented were cited by all the participants in the study suggesting strong agreement with these factors as facilitative of successful transfer.

**Table 2:** Talent Transfer Environment.

Higher Order Theme	Lower Order Theme	Raw Data
Talent transfer environment	Positive learning environment (N = 7)	Talent transfer system adapted to athlete’s needs
		Positive learning environment
		Individual attention
		Individual coaching
		Encouraging athletes’ input
		The lack of a structured pathway
	Time frame of talent transfer process (N = 7)	Athletes were given sufficient time, resources, and attention to adjust to the transfer sport
		No early pressure for results

**Table 3:** Individual Factors Underpinning Talent Transfer.

Higher Order Theme	Lower Order Theme	Raw Data
Individual Factors Underpinning Talent Transfer	Previous sporting experiences (N = 7)	Understood what it takes to compete and train at a high level
		Transfer of learning from donor to transfer sport
		Past experiences gave athlete confidence in own ability
		Ability to create an effective learning environment in transfer sport
	Physical and physiological characteristics (N = 7)	Generic athletic ability was important
		Did not fit the ideal physical profile for transfer sport
		Physical capabilities compensated for lack of technical proficiency in transfer sport
	Psycho-behavioral factors (N = 7)	Commitment and determination
		Confidence
		Coping skills
		Focus and discipline
		Goal setting
		Motivation
		Realistic performance evaluation

### The TT Environment

#### Positive learning environment

Haskell (2001), amongst others [24] emphasize the importance of a holistic approach to talent development, with the former suggesting that transfer of learning needs to make reference to both the learner and the environment. Given this, it was unsurprising that participants described several positive features associated with the TT environment that facilitated and accelerated their progress. As mature athletes, they appreciated the individualised learning environment and suggested that this “fast tracked” their initial development in the sport. Participant 4 suggested that:

The structure really helped. I was really lucky with the people I had around me, I had a lot of backup, and they really encouraged me, helped me progress.

Supporting De Corte’s (2003) assertion that productive learning during the transfer process is facilitated by good teaching, participants attributed much of their success to the individual coaching they received and the individualized system created for them. Participant 4 described the importance of this coaching focus:

It was important that a lot of focus was put into me. I needed that attention and specific individual support at that stage. I was lucky in that I was one of two athletes they were depending on to reach the Olympic Games.

This learning environment maximized previous experiences by encouraging athletes’ input into training with coaches facilitating the transfer athletes to become “agents of their own learning and transfer” (p. 144) [32]. Participant 5 described how:

There was that trust, I was really dependent on what I was being told to do from a technical perspective. But I was also able to impart a lot of information about what I needed as an athlete from a coach, for a good training environment and how I need to train.

Participants also identified a number of negative aspects of the TT environment, unsurprising given the ad-hoc nature of the informal transfer process. For over half of the participants, the lack of a structured pathway for late entrants made it difficult to progress and seek out opportunities for development. Specifically, they suggested that a lack of coherence and consistency in the opportunities offered to TT athletes slowed down their development within an already “time-pressured” pathway. For example, Participant 2 suggested that:

It is taken for granted that you would have gone through the ranks and learned the ropes but if you don’t they don’t really facilitate you.

#### Time frame of TT process

Perhaps in contrast to formal TT initiatives that typically have a short selection and confirmation period, the athletes in this study described how a long-term approach characterized their TT environment. Participants felt that there was *no early pressure for results* when they transferred to the new sport. In fact, all the participants described relatively poor early performances before they adapted to the demands of the activity. Illustrating this, participant 2 described her early experiences in the sport “I mean if you had seen my first couple of performances then you would have given me no chance! But the more I did it the better I got!” The lack of pressure to “get it right straight away”, and the suggestion that the athletes were given sufficient time, resources, and attention to adjust to the transfer sport before they were expected to perform, was cited as key to their success. Participant 4 was typical in how she valued this environment:

One of the biggest things was that there was no pressure from anyone, which was good. I was given as much time as I needed and there was no cut off point that said if you don’t perform by then you are cut.

Interestingly, significant differences in the timeframe needed to reach elite levels were evident. One participant won her first world championship medal within six months of taking up the sport whereas another only broke onto the world stage after two years of competing. It is also interesting to note that only two of the participants would have met the 15–25 years of age criterion associated with formal TT initiatives [6]. The range of ages at which transfer occurred is perhaps indicative of the complexities of development in different sports and the idiosyncratic pathways that typify development in sport [8]. These findings suggest that an understanding and empirical evidence base of the time zones for effectively targeting and developing TT athletes is required as, without this understanding, potentially talented athletes may either be overlooked during the initial selection process or prematurely cut from talent confirmation phases [12].

### Individual Factors Underpinning Talent Transfer

#### Previous sporting experiences

All participants described how their previous experiences helped them adjust and thrive in a new environment. The deliberate practice and experience [33] accrued during their previous sporting experiences facilitated their success in the transfer sport. Interestingly, and despite the emphasis on physical and physiological attributes in formal TT processes [12], participants suggested that their experience in the donor sport helped them understand what it takes to compete and train at a high level in terms of quality training, rest, and recovery, as described by Participant 6:

As an experienced athlete I knew how to push myself, how to do things at the right intensities, I knew what works for me.

This finding reflects Haskell’s (2001) assertion that, every time progress occurs, previous learning is used as a building block; notably our sample were supported by coaches who effectively promoted learning in the transfer sport by building on experience in the donor sport. Participants’ prior experiences at the top level of sport enabled them to transfer learning from donor to transfer sport by making links to prior learning within their own lived experiences [11]. For example, skills and strategies in the transfer sport were adapted to accommodate participants’ prior sporting experiences. Participant 7, a combat sport athlete, described this process:

One thing we worked on was adapting the [donor sport] technique I had to [transfer sport] rather than starting from scratch. We never did the basic textbook stuff, the baby steps, we jumped straight into it because of where I had come from.

In this manner, prior experiences in sport can be understood as preparation for future learning [34] in that participants were confronted with challenges that had personal meaning and were representative of the tasks they would encounter in the transfer sport [32]. These results suggest that coaching style and agendas should usefully vary for TT athletes from that within the “normal” pathway.

#### Physical and physiological characteristics

It was interesting that five out of the seven participants suggested that they did not fit the ideal physical profile for their transfer sport and all pointed out peers who possessed, in their opinion, ideal physical attributes but did not make it to the top of their sport. For example, Participant 4, an Olympic medalist, described how her coach used to talk about her to other athletes: “Look at [name], she is too small to be an international in [transfer sport] …if she can make it, anyone can!” In this regard, participants described how they compensated for physical or physiological deficiencies by strengths in other areas such as work ethic and commitment. Indeed, Participant 3 was able to describe a peer who fitted the physical and physiological profile for success, but who failed to convert that potential:

[Name] had been in [transfer sport] for years and had some of the best numbers in terms of V02, lactates, the works. But she was never able to transfer those scores into a race…you could take her to the track or put her in a lab and she would have brilliant numbers but numbers is one thing and racing and sticking with it is another.

Without an appreciation of this multiplicative approach, there is a risk of underestimating the importance of environmental and intrapersonal factors and overestimating the magnitude of genetic variables [35]. These findings suggest that the focus on the search for an ideal physical and anthropometric profile may lack validity and might even fail to select athletes who could ultimately achieve at the highest level [17]. Interestingly, a number of participants (n = 3), familiar with current (i.e., formal) TT initiatives, described how they would not be picked for these initiatives based on their physical profiles. As one example, Participant 3 recounts her perceptions of this process:

When I first came into [transfer sport] as a transfer athlete, if I did one of their TI (talent identification) days I would barely have made a national junior squad but that year I medaled in three European cup races and that was a bronze, silver and gold in decent races.

The consensus amongst participants was that their generic athletic ability, rather than particular strengths or weaknesses, was more important, with Participant 2 going as far as suggesting that “the sport didn’t matter, I think I could have been successful in any sport.” Indeed, in the current study and elsewhere in the literature [8], there are many cases of successful TT where obvious physiological or motor skill crossover is not evident (e.g., skiing to rowing, basketball to rowing). Supporting Bransford and Schwartz’s (1999) definition of transfer in terms of preparation for future learning and not in terms of identical elements, these results suggest that it would be sensible for TT initiatives to consider early in the identification and coaching process the full range of factors that individual athletes bring into transfer sports rather than attempting to focus on rigid and predefined profiles.

#### Psycho-behavioral factors

All participants described a motivation and desire to compete at the highest level in sport as the factors underpinning their decision to initially transfer to another sport. For example, Participant 3 noted that:

You are constantly thinking I have achieved all I can in this sport, what is next? It is important not being afraid to say that I want to go to a sport where I can achieve more, I wasn’t happy with just being in the pack, I wanted that feeling of being the best and competing at that level.

The participants also described the motivation to learn new skills and be challenged in the performance environment as key factors that facilitated their development; participant 1 describes how:

I love trying new things and being really rubbish at them but feeling my way through and learning it, and working harder and trying to get better. I relished that environment and being in an underdog situation…being really under pressure and having a positive challenge to work towards.

Commitment and dedication, factors that had already underpinned their success in their donor sport, enabled participants to continue to invest the requisite hours into training and to make the necessary sacrifices to succeed in the transfer environment. For example, Participant 2 stressed the importance of these characteristics:

As soon as I get something in my head that I am going to do then that is all I think about. I become completely consumed by it and that is all I want to do. That was the same with [donor sport] and [target sport]—I completely immersed myself in that and did everything to make it happen.

Indeed, the confidence participants had gleaned from their achievements in their donor sport was a key factor that smoothed their transition and enabled them to set, and adhere to, ambitious goals from the very start. Participant 4 typified the approach used by this cohort of athletes:

I believe in setting high targets and maybe just beyond what is achievable and then either you just miss them and then you do ok, or by fighting to reach those targets you draw more out of yourself and you get better and better. I think we aimed to reach the very top. It made it tough because it was constantly pressure, and the feeling of being against the clock but that was just the way it was.

Furthermore the ability to focus and stay disciplined were cited as particularly important during the transfer process. Participants all described how a range of coping skills developed and refined during their initial involvement in sport and later deployed in the transfer sport enabled them deal effectively with the challenges encountered. Participant 5 suggested that both problem and emotion focused coping skills helped her thrive in a difficult situation:

I don’t think I would have coped if I had met him [coach] when I was younger. I think I was mentally strong enough to deal with it then. You had to have a lot of mental strength when you did get to that stage. You had to be able to cope with him, be able to put things into perspective, separate things out.

In a similar manner, participants’ previous experiences of competing at an elite level equipped them with the skills to make realistic performance evaluations and thus maximize learning. Of course, a certain amount of physiological and/or motor control overlap would benefit the transfer process but it is important to stress that, although further evidence is needed, mechanisms behind TT and the factors responsible for causing success are multiplicative [17] rather than the simpler, uni-dimensional approaches which seem to be currently employed.

## General Discussion

TT initiatives offer a unique pathway for athletes to continue participation in sport at the highest level and, by doing so, potentially increases a nation’s capacity for success at major sporting championships. Indeed, there are a number of examples of successful talent transfers athletes competing successfully at the highest level, albeit there are equal or even greater numbers of ‘success stories’ apparent from informal rather than structured (and expensive!) formal TT programmes [8]. As such, this study represented an initial exploration of the factors that TT athletes perceived as supporting their success in an effort to understand the athlete characteristics, learning and transfer tasks, and transfer context which should be exploited if this process is to be optimized [32].

With a predominant focus (certainly during initial screening) on physical, physiological and anthropometric capabilities, many formal TT initiatives may be criticized for treating knowledge and ability as a static property and adopting inappropriately narrow criteria for successful transfer [36]. The athletes in this study emphasized the importance of environmental and psycho-behavioral factors as key to their TT success. Unfortunately, these seem to be rarely considered within current formal TT identification processes beyond a cursory inclusion in athlete specification: “athletes must be mentally tough and competitive” is a typical, though rarely operationalized, requirement during initial recruitment. Reflecting this, it may be more appropriate to reconceptualize TT as the broad, productive, and supported use of acquired knowledge, skills, and motivations as opposed to the direct and sequestered application of skills from one situation to another. Therefore, and reflecting the factors highlighted by the athletes in this study, it is important to move beyond the direct application theory of transfer [34] towards a focus on assessing athletes’ abilities to learn in new, knowledge-rich environments [32]. In this regard, these results suggest that the role of psycho-behavioral factors, self-regulation and the environment must be considered [37] in fostering the use of knowledge and skills that should facilitate effective transfer [38].

The range of factors cited by athletes as facilitative of successful transfer incorporated the interaction of a range of factors including situational factors and psychological skills. This dynamic and multidimensional approach to talent development [39] suggests that greater effort should be placed on identifying an individual’s capacity to learn rather than measuring what has already been learned. Of course, there is no disputing that physical and anthropometric factors play a role in elite sport; however they are clearly not the only or, perhaps, even the most important factor. Indeed, the current findings certainly suggest that there is a need to consider and further explore the individual skills and characteristics required to meet the challenges faced in the transfer sport, along with an emphasis on appropriate coaching environments and timeframes associated with TT [40]. Given the exploratory nature of this study (the second in the field as far as we are aware), it is important that future research is conducted that tests the generalizability of these findings in other contexts (team sports, for example). It may well be that there are other reasons underpinning TT. For example, the significant transfer between summer and winter sports is a phenomenon likely explained by cultural, topographical and pragmatic factors [8]. Future studies should adopt a longitudinal approach to further examine the TT process and the factors that facilitate successful transfer between sports. Indeed, the rich picture of the TT process described by the participants, including contradictions, emphasizes the complexity of the picture and, we would suggest, challenges the ‘one size fits all’ system that formal TT programs adopted. Of course, future research that compares these findings to other TT initiatives (e.g., non-successful TT athletes, formal TT athletes) would shed some light on the factors that underpin this process.

Of course, one could criticize our results by questioning the nature of the sample; namely, that all were informally recruited or self-initiated in their change (i.e., informal TT), rather than moving through the formal processes that exist today. It may be that, for a variety of reasons (including the need for quick results or ever increasing world class standards in sport), our findings lack relevance to today’s challenges. We acknowledge and state this caveat and await empirical evidence to support it. This point notwithstanding, however, it would have seemed to us worthwhile to check with successful transfers as a *first* step in developing formal TT models! Notably, none of our sample had been questioned as a part of the development process for the talent transfer systems which are currently running despite their high profile successes.

We hope our results will be useful to sporting bodies as they consider and refine TT methodologies as well as provoking a critical review of, and debate about the scientific and empirical basis for these projects. Once again, a need for evidence-based practice to support the employment of these initiatives would seem a logical next step.
